# Tissue Immune Cells Fuel Obesity-Associated Inflammation in Adipose Tissue and Beyond

**DOI:** 10.3389/fimmu.2019.01587

**Published:** 2019-07-17

**Authors:** Rui Liu, Barbara S. Nikolajczyk

**Affiliations:** ^1^Department of Pharmaceutical Sciences, College of Pharmacy, University of Kentucky, Lexington, KY, United States; ^2^Department of Pharmacology and Nutritional Sciences, Barnstable Brown Diabetes and Obesity Research Center, University of Kentucky, Lexington, KY, United States

**Keywords:** tissue-specific T cells, crosstalk, obesity, inflammation, cancer

## Abstract

Obesity-associated inflammation stems from a combination of cell-intrinsic changes of individual immune cell subsets and the dynamic crosstalk amongst a broad array of immune cells. Although much of the focus of immune cell contributions to metabolic disease has focused on adipose tissue-associated cells, these potent sources of inflammation inhabit other metabolic regulatory tissues, including liver and gut, and recirculate to promote systemic inflammation and thus obesity comorbidities. Tissue-associated immune cells, especially T cell subpopulations, have become a hotspot of inquiry based on their contributions to obesity, type 2 diabetes, non-alcoholic fatty liver diseases and certain types of cancers. The cell-cell interactions that take place under the stress of obesity are mediated by intracellular contact and cytokine production, and constitute a complicated network that drives the phenotypic alterations of immune cells and perpetuates a feed-forward loop of metabolic decline. Herein we discuss immune cell functions in various tissues and obesity-associated cancers from the viewpoint of inflammation. We also emphasize recent advances in the understanding of crosstalk amongst immune cell subsets under obese conditions, and suggest future directions for focused investigations with clinical relevance.

## Introduction

We have raised an entire generation of adults who were born into the global epidemic of obesity and obesity-associated complications like non-alcoholic fatty liver disease (NAFLD), type 2 diabetes (T2D) and many cancers. Both the appreciation of the societal costs of obesity and the physiological understanding of obesity-associated diseases have evolved over the decades. The discovery that obesity generally induces low-level but chronic inflammation in classical metabolic tissues and beyond revolutionized the concept that obesity was a strictly metabolic disorder: obesity should also be regarded as an inflammatory disease with characteristics that are distinct from classical inflammation caused by infection (1–3). The inflammatory response in obesity initially results from excessive nutrient accumulation and disturbed metabolic homeostasis. These lead to alterations in amounts and species of various molecules that function as endogenous ligands to activate immune cells. Metabolic regulatory cells such as adipocytes also secrete inflammatory cytokines and chemokines. Although concentrations of the so-called “adipokines” like IL-6 are generally modest compared to amounts secreted by immune cells, these products recruit immune cells to adipocyte-imbedded metabolic tissues over a relatively prolonged time course (1). Initial focus on relatively independent roles of each cytokine or immune cell subpopulation through knockout approaches has led to helpful insights into the role inflammation plays in metabolic derangement. These early studies are now being expanded as the field builds an appreciation of how crosstalk amongst diverse immune cell populations mediates obesity-induced inflammation.

Adipose tissue (AT) is arguably the most commonly studied source of immune-mediated inflammation in obesity. In addition to immune cells, this complex endocrine organ contains diverse cell types such as pre-adipocytes, adipocytes, fibroblasts, and endothelial cells (4). Given the numerous comprehensive reviews of phenotype, function and distribution of tissue-specific macrophages in obesity already published (5, 6), and the likely equal importance of T cells in human obesity (7–9), this review will focus on the participation of T cells in obesity with a brief mention of macrophages. T cell subpopulations and their role in AT inflammation have been studied in various murine models of obesity. A developing theme is the importance of immune cell crosstalk, as measured by the innate immune cells such as the macrophages and dendritic cells (DCs) that regulate T cell responses to AT expansion. However, in contrast to the multitude of (murine, with fewer human) studies on AT-associated immune cells, immune cell populations in other metabolic tissues, including liver and gut, are less understood, despite broad agreement that these organs are also stressed and inflamed in obesity. Although immune cells outside the AT or blood have garnered little attention, they are likely equally important as AT immune cells in metabolic regulation and overall health. Herein we discuss functions of adaptive immune cell subsets in AT, liver and intestine during obesity and obesity-associated cancers, with additional focus on the importance of crosstalk between T cells and antigen presenting cells (APCs).

## Adipose Tissue Immune Cells in Obesity-Associated Inflammation

Normal AT function relies on a considerable amount of oxygen, which generally surpasses the oxygen requirements of other tissues (10). Adipocyte enlargement in response to obesity increases the distance between AT cells and the AT vasculature and thereby leads to hypoxic conditions (10–12). Hypoxia, like insulin, activates expression of hypoxia-inducible factor 1 (HIF-1) in adipocytes, which counters the negative consequences of adiposity (12). However, chronic hypoxia ultimately results in cell death, in part by maintaining and amplifying inflammatory signals that concomitantly regulate differentiation of immune cell population (13–18). One clearly detrimental outcome of chronic AT hypoxia is fibrosis (19). A second outcome is that macrophages infiltrate AT to scavenge dead and necrotic adipocytes in the involved fat depot, which subsequently amplify a local inflammatory response by producing IL-6 and TNF-α (20, 21). Macrophages constitute a large proportion of immune cell populations in AT and have been commonly investigated during obesity. A shift in frequency from a simplistically designated anti-inflammatory M2-like (F4/80^−^) to a pro-inflammatory M1-like (F4/80^+^) macrophage phenotype is considered a main contributor to AT inflammation in obesity, since the numbers and frequencies of M1-like macrophages correlate with insulin resistance (IR) (22). Macrophages are an important source of AT inflammation (23–25), but numerous changes in T cell subpopulations in the AT also track with the development of obesity and IR in animal models. Most research has focused on T cells bearing the αβ T cell receptor (TCR), which includes both CD4^+^ and CD8^+^ T cells. These changes broadly include increased CD8^+^ T cell activation and subset-specific changes in CD4^+^ T cells: fewer regulatory T cells (Tregs) and Th2 cells, and a higher frequency of Th1 and Th17 cells, all hallmarks of a shift from an anti-inflammatory to pro-inflammatory milieu. Many studies indicated that these subset changes relied on the production of cytokines and chemokines, which are generally produced by or regulate specific T cell subsets as well as macrophages. However, specific experimental data (for example, labels that track cells) have not always been used to precisely discriminate between recruited and tissue-resident immune cells. Definitive work has shown that M1-macrophages are recruited to AT in obesity (26, 27), whereas both anti-inflammatory Tregs and pro-inflammatory γδ T cells are AT resident (28–30). Distinction between resident and recruited cells from other immune subsets have not generally been studied, apart from parabiosis studies showing most subsets can recirculate.

### Adipose-Associated Th1 Cells Promote Obesity-Associated Inflammation

The frequency of Th1 cells in human visceral adipose tissue (VAT) and subcutaneous adipose tissue (SAT) depots of obese but non-T2D human subjects is 10–20-fold greater than that of Th2, Treg or Th17 cells. Th1s were more frequent in VAT compared to either SAT or peripheral blood mononuclear cells (PMBCs) of each individual tested (31). Frequency of VAT and/or SAT Th1 cells robustly correlated with traditional inflammatory markers in plasma, including IL-6 and hsCRP (31), and also with circulating Th1 frequency (31). Comparison of VAT or SAT of morbidly obese and lean subjects also showed that gene expression of the Th1 transcription factor T-bet was similar (32). A third study showed that IFN-γ mRNA expression in SAT correlated with waist circumference of T2D patients (33), and independently suggested that activated pro-inflammatory T cells are present in AT. Although higher IFN-γ expression can be attributed to CD8^+^ T cells, CD8s are relatively rare in AT (33). Bioactive IFN-γ is instead likely produced by CD4^+^ Th1 cells through HLA-DR^+^ (human MHC Class II)-mediated antigen presentation from nearby macrophages, although NK cells are another theoretical source of IFN-γ. Generally speaking, IFN-γ production by Th1 cells can be activated by HIF-1 under conditions of tissue hypoxia, which also increases phosphorylation and thus activation of STAT3, a master Th17 transcription factor. These data are consistent with demonstrations that Th17s may also fuel AT inflammation, as discussed below, although applicability to obesity-associated inflammation remains unknown (15). In contrast to mouse studies (34), correlations between AT Th1 frequencies and IR in obese humans are inconsistent (31, 33), perhaps due to analysis of different AT compartments, the variation in enrollment criteria for obese subjects, or reliance on different markers to define cellular subsets. In high fat diet (HFD)-fed mice, Th1-polarized T cells generally accumulate at epididymal white adipose tissue (epiWAT), in part due to increased expression of RANTES (CCL5), and coincident with the resolution of epiWAT remodeling (35).

### Adipose-Associated Th2 Cells Attenuate Obesity-Associated Inflammation

Th2 cells are a relatively rare population in AT compared with other CD4^+^ T cell subsets, although the frequency of Th2 cells is significantly higher in SAT (but not VAT) as compared to PBMCs (31). In contrast to Th1 cells, Th2 cells provide beneficial effects in maintaining glucose homeostasis in *ex vivo* studies or animal models, probably by secreting IL-4 and IL-13. Th2 cells in human VAT inversely correlated with plasma hsCRP concentration, a stress indicator that also suggests systemic inflammation. In both SAT and VAT, frequency of Th2s, but not other CD4^+^ T cell subsets, also associated with systemic IR (31). A second clinical study similarly indicated that low Th2 frequency in VAT of obese subjects is linked to systemic inflammation (32). Mouse studies more definitively show concordance between reduced Th2 frequency in AT and loss of their context-dependent anti-inflammatory functions. The percentage of CD4^+^GATA-3^+^ Th2 cells were significantly lower in VAT of 16-week HFD-fed mice compared to leans, but without alteration in absolute numbers of Th2 cells per weight of VAT (34). Anti-obesity effects of Th2s were indicated by CD4^+^ T cell transfer into Rag1^−/−^ diet-induced obese (DIO) mice, which prevented weight gain and improved insulin sensitivity as a likely outcome of increased (STAT6-dependent) Th2s (34). The overall consensus amongst studies is that the reduction of Th2 cells in AT disrupts the original non-inflammatory balance between Th1 and Th2 cells, leading to a Th1-dominated pro-inflammatory environment in obese mice that is also evidenced, albeit variably, in people (34).

### Adipose-Associated Th17 Cells Support Obesity-Associated Inflammation

Numerous studies have found higher frequency and/or function of CD4^+^ Th17 cells in human VAT, as well as more broadly (36–38). Mechanisms that increase Th17 cells in people likely include adipokines, as evidenced by the demonstration that supernatants from incubated omental AT, one type of VAT, from morbidly obese (metabolically uncharacterized) subjects activated IL-17 production by circulating memory CD4^+^ T cells (39). APCs also impact IL-17 production as indicated by demonstrations that CD11c^+^CD1c^+^ DCs activated IL-17 expression in SAT of obese patients, and that adipocytes may activate Th17s in VAT (40). Purinergic signaling can drive Th17-associated responses including activation of the master transcriptional activator of Th17s, RORγt, higher expression of IL-23R to promote Th17 survival, and IL-17 secretion in VAT of metabolically unhealthy obese subjects (41). The presence of IL-17 neutralizing antibodies markedly reduced the inflammatory response of CD45^+^ T cells in human AT, indicating that Th17s may fuel a feed-forward loop of AT inflammation (39). Although CD4^+^ T cells are the vast majority source of IL-17 in PBMCs (42), γδ T cells and innate lymphoid cells (ILCs) may be equally/more important sources of tissue IL-17. Therefore, changes in IL-17 production cannot be simply attributed to changes in Th17 frequency (28, 43). Parallels between Th17s in human and mouse are not entirely consistent. Although earlier studies discounted the importance of Th17s in AT of obese mice (34), subsequent reports showed that DIO mice have enlarged pool of IL-6-dependent Th17 cells, and that IL-17^+^ cells increased in AT of obese compared to lean mice (44). Mechanistic studies on the bias toward Th17 differentiation in obesity implicated Acetyl-CoA carboxylase 1 (ACC1), which activates fatty acid synthesis in memory CD4^+^ T cells and controls the transcriptional activity of RORγt to activate IL-17 gene expression (45). Recent work from our lab highlighted additional mechanisms of increased Th17 frequency in T2D: higher IL-17F frequency was induced in cells from lean/euglycemic subjects with a combination of experimentally-induced mitochondrial changes coupled with long chain fatty acid metabolite challenge (46). Independent work showed obese mice, due to either HFD or the ob/ob mutation, had increased Th17 responses triggered by CD11c^+^ DCs (40), even amidst the T cell developmental defects in ob/ob (and db/db) mice. AT hypoxia may also contribute to Th17 differentiation in obese mice based on the action of HIF-1α (17). HIF-1α regulates the Th17/Treg ratio by enhancing transcriptional activation of RORγt while suppressing Treg development through promoting proteasomal degradation of Foxp3 (17). HIF-1α also regulates Th17 cells outside the context of obesity (18, 47), suggesting that additional mechanisms may link HIF-1 and Th17 in obesity.

### Adipose-Associated Tregs Counter Obesity-Associated Inflammation

Tregs are an anti-inflammatory T cell population that are relatively frequent in lean subjects, but with the progression of obesity, the frequency of AT Tregs declines in people and in mice. Tregs occupy 5–20% of total CD4^+^ T cells in the human peripheral lymphoid compartment, but the percentage of Tregs in human AT remains controversial, perhaps in part due to general paucity of immune cells in typically sampled human VAT compared to mouse epiWAT. The fraction of Tregs in mouse VAT is larger compared to other locations such as spleen, lung and liver (29, 48). Adipose resident Tregs in lean individuals differ from conventional Tregs based on both gene expression profiles and TCR repertoires (9, 29, 49, 50). VAT resident Tregs in leans produce IL-10, which inhibits the TNF-α-induced expression of IL-6, RANTES, SAA-3 and MMP-3. IL-10 also restores IRS-1 phosphorylation and consequently rescues the expression of GLUT4 after TNF-α treatment in adipocytes (29). In VAT, IL-33 regulates Tregs through the IL-33 receptor ST2, and IL-33 treatment reversed the inflammatory status generated by reduction of VAT Tregs in obese mice (51). ST2 ablation in mice resulted in a significant loss of VAT and even of rare SAT Treg populations (49). However, independent analysis of ST2 showed IL-33 also induces innate lymphoid cell type 2 (ILC2) activation and eosinophil accumulation in mouse VAT, suggesting these mechanisms parallel anti-inflammatory effects of VAT-associated Tregs (52). Phenotypic differences in VAT Tregs compared with Tregs from spleen and lymph nodes also include PPARγ expression, which collaborates with Foxp3 to impart naïve CD4^+^ T cells with VAT Treg characteristics. This finding likely explains the mechanism of the anti-inflammatory effect of PPARγ-targeting thiazolidinedione in AT (30). Regulatory effects of HIF-1 on Treg development may differ under obese conditions (17), given that HIF-1 activation upregulates the expression of Foxp3 to promote Treg proliferation and thus enhance immunosuppressive activity (13, 14), seemingly conflicting with actions of HIF-1α outlined above (17).

### Adipose-Associated CD8^+^ T Cells Promote Obesity-Associated Inflammation

CD8^+^ T cells are relatively rare compared to CD4^+^ T cells in VAT of humans (33). VAT houses higher numbers of CD8^**+**^ T cells than SAT in obese compared to lean subjects, and the CD8^+^ cell numbers positively correlated with individuals' BMIs (53). The numbers of both memory and effector CD8^+^ T cells per AT mass are higher in VAT than SAT, but numbers of naïve CD8^+^ T cells between VAT and SAT of obese patients with BMI over 40 kg/m^2^ were indistinguishable (53). Consistent with AT CD8^+^ T cells in the morbidly obese, the percentage in the lymphocyte fraction is higher in VAT than SAT of overweight subjects (54). Jointly, abovementioned results indicate CD8^+^ T cells accumulate in specific AT compartment in obese humans. Studies in mice showed that CD8^+^ T cell activation is one of the earliest events in the inflammatory response to obesity, preceding M1-like macrophage activation/infiltration in AT (55). CD8^+^ T cell influx is associated with the increased expression of HIF-1 under hypoxic WAT environments in both DIO and ob/ob mice (16). The demonstrated impact of HIF-1 on the expression of pivotal transcription, effector, and costimulatory/inhibitory molecules of CD8^+^ T cells suggest mechanisms of HIF-1 CD8 regulation that will require validation (56). Accumulated epiWAT CD8^+^ T cells produce perforin and granzyme that in turn may help clear compromised adipocytes in AT crown-like structures. Because the number of circulating CD8^+^ T cells is not altered in parallel, the data suggest tissue-specific activation of CD8^+^ T cells instead of recruitment to AT during obesity (57). CD8^+^ T cells appear to interact with adipocytes and macrophages in obese AT to trigger an inflammatory cascade via an interactive circle (57). A recent study raised the possibility that isolevuglandin-containing antigens presented by M2-polarized macrophages to CD8^+^ T cells in the AT of DIO mice may preferentially expand cognate TCR clonotypes (58). Thus, obesity-associated antigens may select and modify the TCR repertoire in the AT and thereby alter immune responses at adipose depots.

### INKT Cells Protect Against Obesity-Associated Inflammation

Invariant natural killer T (iNKT) cells are predominantly cells that express an invariant TCRα chain coupled with a limited/defined group of TCRβ chains. Unlike traditional αβ T cells, which recognize antigen peptides in the context of MHC Class I or II molecules and are activated by stimulatory signals from CD3/CD28 and other co-receptors, iNKT cells recognize glycolipid antigens bound to a non-classical MHC molecule CD1d that presents, for example, α-galactoceramide (59). Upon activation by α-galactoceramide, iNKT cells produce cytokines such as GM-CSF, TNF-α, IFN-γ, IL-4, IL-10 and IL-13 through differentiation into subsets that parallel functions of the various CD4^+^ T cell subsets (60–62). iNKT cells are enriched in human omentum, but with lower frequency in omental tissue of patients with severe obesity compared to leans (60). Although iNKTs are present in human AT, functional investigations of AT iNKTs were conducted in DIO mice. Lipid metabolites from adipocytes that have been stressed by excess fatty acid accumulation in obesity may provide CD1-presented antigens that activate iNKT cells to decrease AT inflammation, based on evidence that adipose CD1d deletion prevented the anti-inflammatory effect of iNKTs (63). CD1d is expressed on multiple APCs, but the unexpected demonstration that adipocytes express CD1d more than traditional MHC molecules suggests direct interaction between adipocytes and resident/infiltrating iNKTs (64). Adipose iNKTs may help sustain Tregs through IL-2 supply, consistent with demonstrations that CD1d knockout mice have lower IL-2 production in AT, which associated with fewer adipose Tregs during the short-term HFD feeding (65). However, the general ability of Tregs to efficiently scavenge low concentrations of IL-2 from the surrounding milieu more effectively than other CD4^+^ T cell subsets (due to high CD25 expression) undermines this interpretation of iNKT/Treg interdependence. Fewer iNKTs associated with reduced M2-like macrophages at early time points, amidst M1-like macrophage accumulation in obese AT (66). This macrophage shift exacerbated adipose inflammation in part by increasing IL-6 and IL-1β (63). The regulatory role of iNKTs on adipose macrophages and Tregs may also be due to IL-10 production (67).

### Adipose-Associated γδ T Cells Increase Obesity-Associated Inflammation

γδ T cells are a T cell lineage that is developmentally distinct from αβ (CD4^+^ and CD8^+^) T cells based on expression of the γ and δ genes for the surface TCR. An understanding of γδ T cells in human obesity is very limited: the number of γδ T cells is lower in obese compared to lean AT, and negatively correlates with BMI (68). Roles for human γδ T cells in obese AT must therefore be imputed from studies in mice. TCRδ ^−/−^ mice, which lack γδ T cells, were more insulin sensitive compared to wild-type mice in response to HFD (69, 70), indicating γδ T cells promote obesity-associated inflammation. This result conflicts with the speculation that γδ T cells protect from obesity-associated inflammation in humans from the limited data outlined above. Numerous mechanisms explain the pro-inflammatory origins and functions of γδ T cells in obese mice. Gain- and loss-of-function studies showed CCL2 and IL-6 expression associate with the frequency of γδ T cells, indicating these cells may originate downstream of primary immune cell and/or adipocyte changes in obesity. Functions of γδ T cells in obesity are perhaps better understood than the time at which these cells contribute to AT inflammation. γδ T cells produce IL-17 through mechanisms independent of TCR signaling, with innate cell-like activation through pattern recognition receptors, cytokine or chemokine receptors. γδ T cells thereby further activate CD4^+^ T helper cells in obesity (43). Tissue-resident IL-17A-producing γδ T cells also negatively regulate the Treg population in AT (28). Finally, TCRδ knockout mice had less CD11c^+^CD206^−^ M1-like and TNF-α^+^-macrophage infiltration in epiWAT in response to HFD, suggesting multiple direct and indirect functions of γδ T cells in obesity. Although current evidence concurs that γδ T cells promote inflammation in AT during obesity, further investigations are required to determine whether γδ T cells have immunosuppressive roles in regulating DC dependent-T cell proliferation in obesity, as reported under other conditions (71). γδ T cells may also have cytolytic functions that are beneficial for countering malignancy in obesity, similar to CD8^+^ T cells (72). We speculate that reducing γδ T cells in AT during obesity may ameliorate inflammatory actions that trigger broader T cell response and adipocyte apoptosis.

### B Cells Pleiotropically Contribute to Obesity-Associated Adipose Tissue Inflammation

B cells promote obesity-associated metabolic disease through their ability to infiltrate the expanding AT, produce cytokines, and/or to produce autoreactive antibody (IgG) to trigger local inflammation (73–75). However, some studies suggest protective roles of AT-associated B cells in obesity-potentiated inflammation. This seeming contradiction is partially resolved with the appreciation that B cells, like T cells, fall into multiple subsets that differ functionally. Major B cell subsets include B1 and B2 cells, the latter being further subdivided based on the ill-defined regulatory B cells (Bregs)/B10 subset, characterized by production of IL-10 and any number of surface markers (76, 77). Each of these B cell subsets has been implicated in obesity-associated AT inflammation, mainly in studies from mice. B2 B cells in VAT produced IgG that was pathogenic, while omental AT-enriched B1 B cells generate so-called “natural antibody” (IgM) that is generally anti-inflammatory. The importance of natural antibody in AT inflammation was indicated by data showing activated B1 B cell-derived IgM production inversely correlated with circulating MCP-1 (CCL2) levels in obese people (78). MCP-1 is associated with Th2 response and also regulates macrophage chemotaxis, hence perhaps playing an important role during chronic inflammation. These data were consistent with phenomena observed in DIO mice, that B1 B cells blunt M1-like macrophage-mediated inflammation, in part by reducing TNF-α and MCP-1 in AT (78).

**Figure 1:**
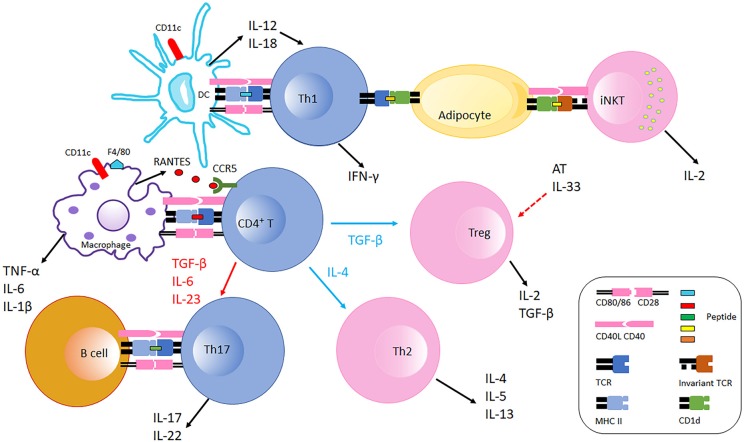
Immune cells interaction in adipose tissue in obesity. Obese AT shifts macrophage polarization to the M1 phenotype (F4/80^+^), macrophages recruit CD4^+^ T cell by producing RANTES which is the ligand of CCR5 expressed on T cells. With the stimulation of fate specifying cytokines, CD4^+^ T cells differentiate into different effectors including Th1, Th2, Th17, and Treg cells. CD11c^+^ DCs secret IL-12 and IL-18 which further polarize CD4^+^ T cells to Th1 cells in AT. Th1 cells interact with adipocytes through MHC Class II molecules suppressing AT IL-33 production from unspecified cellular sources, which promotes Treg proliferation. Adipocytes also communicate with iNKT cells via CD1d molecules expressed on adipocytes inducing iNKT cells to produce IL-2 and other cytokines that generally counter AT inflammation. B cells and Th17 interaction is also likely mediated by MHC Class II molecules and costimulatory receptors, which facilitates IL-17 and IL-22 production. During obesity, AT Th2 and Treg populations decline, and the balance is thus skewed to pro-inflammatory with increased Th1 and Th17 populations. Typical pro-inflammatory T cells in AT are colored blue, and anti-inflammatory T cells in AT are colored pink.

The mechanisms driving the production of autoantibodies that cause glucose intolerance, and the links between these antibodies and AT inflammation are not detailed (73). The important question of putative autoantibody specificity also remains unresolved, although this query is technically reasonable to address given that antibodies can purify cognate antigens. Regardless, adoptive transfer of SAT B cells from lean mice into DIO mice reduced CD44 and IFN-γ expression in CD8^+^ T cells, and apparently decreased T*nf* and C*cl2* expression in SAT and epiWAT (79). This effect was not observed following adoptive transfer of B cells from IL-10 knockout mice, suggesting that Bregs suppress established AT inflammation. Because Bregs in AT appear to have anti-inflammatory property that is similar to Tregs, comparative study of the relationship and functions of these two regulatory immune cell populations could be an important area of investigation. Precise characterization of B cell subsets in any WAT depot remains elusive. Similarly uncharacterized B cells also accumulate in the brown adipose tissue (BAT) in obesity, although these studies did not further elucidate the phenotype or function (80). Immune cell interactions and contributions to obesity-associated AT inflammation are summarized in Figure 1.

## Liver Immune Cells in Obesity-Associated Inflammation

Visceral lipid accumulation in obesity is especially prominent in the liver, an important organ for cholesterol synthesis and storage of excess fat. Non-alcoholic fatty liver disease (NAFLD) results from excess hepatic lipid, and combines with inflammation and hepatocyte damage to define non-alcoholic hepatitis steatosis (NASH). NASH can progress to hepatic fibrosis, then in some cases, hepatocellular carcinoma (HCC). A definitive NAFLD diagnosis is never straightforward as it requires histological analysis of a liver biopsy, which is painful and can aggravate injury. Heterogeneity of the human liver, even within the same lobe, also challenges a definitive diagnosis. Therefore, life style, medical history, and elevated hepatic enzymes (ALT and AST) are used as surrogates for NAFLD, which may lead to detection later than optimal for the most effective treatment. Current work is focusing on circulating inflammatory markers, including cytokines and chemokines, to offer clues on the transition from NAFLD to HCC.

### T Cell Cytokines as Biomarkers of NAFLD

Differences in T cells and T cell cytokines associate with the shift from NAFLD to NASH. Human NASH is characterized by increased frequency of intrahepatic IL-17^+^ cells along with intrahepatic CD4^+^ T cells. Th17/Treg and Th2/Treg ratios in peripheral blood are higher in NASH as compared to NAFLD (81). Adult NASH patients displayed higher numbers of IFN-γ^+^CD4^+^ T cells and IFN-γ^+^CD8^+^ T cells, and lower numbers of naïve CD4^+^ (CD45RA^+^) and CD8^+^ cells in peripheral blood compared to non-NASH subjects (82). Although consistent stimulation of T cells in liver would typically result in a transition from CD45RA^+^ (naïve) to CD45RO^+^ (memory) subsets that could delineate stages of NAFLD, such outcomes have not been reported from liver biopsies. Pediatric NASH patients had similar increases in IFN-γ^+^CD4^+^ T cells and CD8^+^ T cells, mirroring characteristics of peripheral blood and T cell inflammation in adults (83). Circulating lymphocytes may infiltrate liver due to higher chemokine and chemokine receptor expression in NASH patients with morbid obesity, recapitulating general paradigms of lymphocyte homing (84). Special diets in animal models of NASH shed light on hepatic inflammation, and may support key concepts in human disease. For example, the Th1 response was higher at the presumed expense of lower Th2 responses during chronic liver inflammation in the choline-deficient diet (CDD)-induced murine hepatic steatosis model (85). These findings may, at least on the surface, contrast with the demonstration of a higher Th2/Treg ratio in NAFLD, but can only be rigorously interpreted in the context of full T cell phenotyping. This work was somewhat replicated by a second mouse study, which noted that the overall pool of CD4^+^ T cells decreased in liver in response to CDD induction and was accompanied by HCC development (86). In addition to higher Th1 cytokines during NASH, increased IL-17 production was found during NASH development in methionine-choline deficient (MCD) diet-fed mice (87). Temporal studies indicated that Th17 cells initiate NASH development and contribute to progression, while Th22 cells demonstrated dual roles in cytoprotective and toxic functions: in the absence of IL-17, Th22 cells infiltrated liver to attenuate the hepatic lipotoxicity, but in the presence of hepatic IL-17, Th22 cells are not able to counteract IL-17-mediated liver damage (87). The IL-17/IL-22 ratio in peripheral blood could be used to estimate the prognosis of NASH in the clinic if similar ratio shifts are validated in humans.

### NKT Cell Function in NAFLD

Less plentiful T cell subsets like NKT cells also play roles in obesity-associated liver disease, in part through their ability to regulate hepatic inflammation. Evidence from human liver biopsies and the MCD-fed murine NASH model showed accumulation of NKT cells (not a specified NKT subset) stimulated hepatic fibrosis, exacerbated NAFLD toward NASH and promoted hepatic cirrhosis (88). NKT cells are putatively attracted to the liver through numerous chemotactic agents including enriched lipid antigens from dysfunction of hepatic fatty acid metabolism (89), upregulated Hh-pathway, CD1d, and IL-15 expression in the fibrotic liver (88). Chemokine receptor CCR7-expressing mononuclear cells also recruit iNKT cells to the liver, at least in the HFD-induced NASH model (90). iNKT accumulation aggravates NASH through a pallet of cytokines that change over time: IL-17^+^ and IL-22^+^ iNKT cells emerged at the onset of disease, while IL-4^+^ and IL-13^+^ iNKT increased later in disease pathogenesis (91). In contrast to the pro-inflammatory effects of iNKT cells in NASH, CCR7-dependent iNKT are IL-10^+^, and perhaps thereby reduce net hepatic inflammation (90). Taken together, these data emphasize the importance of NKT subsets and their distinct cytokine profiles and thus pleiotropic function in the hepatic response to metabolic stress.

### Dendritic Cell Function in NAFLD

DC function in human NAFLD has not been tested, but studies in animal models point to complex roles for DCs in obesity-associated liver inflammation. DCs were initially speculated to exacerbate chronic inflammation in liver disease based on their well-documented ability to present antigens and thus stimulate T cells. However, the ability of DCs to present hepatitis B surface (HBs) and core (HBc) antigens was impaired by excess fatty acid accumulation in the HFD-induced NAFLD model, which resulted in less B and T cell activation (92). Although these findings highlight the importance of altered DC function in liver steatosis under conditions of HepB infection, the relevance of impaired viral presentation to DC function in obesity (absent viral infection) was not addressed by this work. Some studies indicated that DCs do not promote obesity-associated liver inflammation. For example, isolated DCs from steatotic liver prevented CD8^+^ T cell activation by phagocytosing debris in response to hepatocyte apoptosis and necrosis in the MCD diet-induced NASH model. DCs also restricted the innate responses initiated by TLR activation in this model and consequently attenuated cytokine production and thus intrahepatic inflammation and fibrosis. These claims of non- or even anti-inflammatory actions of hepatic DCs are countered by findings that these cells proliferate during NASH concomitant with a decrease in Treg numbers, and promoted the proliferation of CD4^+^ effector T cells (Th1, Th2 and Th17 cells) (93). Taken together, these results reflect a multifactorial role of DCs in hepatic biology through interaction with pro- and anti-inflammatory T cells. This interaction emphasizes how bridges between classically defined innate and adaptive immune responses regulate inflammation and thereby hepatic deterioration in NAFLD. However, translating murine data to human NAFLD needs to be done with caution due to, among other issues, lack of definitive and corresponding human DC markers (94), and possible alteration in DC phenotypes during/after the steatotic stage of disease into cirrhosis or even HCC, none of which are well understood.

## Intestinal Immune Cell Subsets and Obesity-Associated Inflammation

Local inflammation in intestine of obese people and mice is subtle compared with that in AT. Regardless, the physiological function and anatomical structure make intestine a prime location for immediate effects of overnutrition and enable the gut to be a reservoir for various antigens that engineer immune cell trafficking to the nearby AT, albeit through poorly understood mechanisms. Apart from trafficking of immune cells from gut to AT, the gut appears to use chylomicrons to deliver food antigens to the gut-associated AT. These antigens may trigger CD4^+^ T cell accumulation and the associated inflammation in the mesenteric AT. Although such antigen delivery did not change weight gain in response to HFD, the resulting inflammation correlated with glucose metabolism defects, indicating that intestinal nutrient absorption can regulate inflammation by controlling T cell accumulation in the fat (95).

Large amounts and numerous species of microbiota, which are both regulators and targets of immune cells, reside in the gut. Adoptive transfer experiments showed that fecal samples transferred from an obese subject to germ-free mice on low-fat diet increased fat mass and body weight in mice. In contrast, fecal samples transferred from a lean twin in parallel studies maintained leanness (96, 97). These findings provided convincing evidence that the gut microbiome regulates metabolic health. Like obese mice, obese people have a different distribution of gut microbe families compared to lean counterparts (98, 99), which can in turn regulate distribution and numbers of gut-associated immune cells. Physical alteration of the intestinal barrier in obesity, as evidenced by an enlarged jejunal mucosa surface in obese compared to lean subjects, may also shift gut immunity as indicated by increased IFN-γ-, IL-17-, and IL-22-producing Th1 and Th17 cells in the lamina propria, and increased CD8^+^ T cell frequency in epithelium. Effects on inflammation outside the gut was not addressed in these studies (100). Duodenal gene expression profiles also indicated more intestinal inflammation in obese compared to lean people as measured by more IFN-γ and IL-1β in the former (101). An HFD-induced Th17 cell population in different intestinal segments has been reported by several obesity studies in animal models (102–104). However, as a note of caution, conclusions on roles for Th17 in obesity-associated intestinal inflammation must be tempered due to demonstrations that confounding factors including stage of obesity, genetics, gut microbiota and husbandry conditions may lead to contradictory results in mice (98, 105, 106). Tissue-specific immune cell functions are summarized in Table 1.

**Table 1:** Tissue-specific immune cell functions during obesity.

		**Mouse**			**Human**		
**Immune cells**	**Function**	**AT**	**Liver**	**Intestine**	**AT**	**Liver**	**Intestine**
M1-Mϕ	+	/	/	/	/	/	/
M2-Mϕ	−	/	/	/	/	/	/
DC	#	/	NAFLD, NASH (HFD, MCD)	UNK	/	UNK	UNK
B cells	+	^*^SAT, epiWAT, VAT	UNK	UNK	UNK	UNK	UNK
NKT	#	− (HFD)	+ NASH, HCC (MCD, CD-HFD)	UNK	−omental AT	# NASH	UNK
CD8^+^ T	+	epiWAT (HFD)	NASH, HCC (CD-HFD)	UNK	VAT	UNK	epithelium
Th1	+	epiWAT (HFD)	NASH (CDD, MCD)	UNK	VAT, SAT	UNK	lamina propria
Th2	−	VAT, SAT (HFD)	^*^NAFLD, NASH (HFD, CDD)	UNK	VAT, *ex vivo*	UNK	UNK
Th17	+	AT (HFD, ob/ob, db/db)	NASH (MCD)	colon, ileum (HFD)	VAT	NASH	lamina propria
Treg	−	VAT (HFD)	UNK	UNK	UNK	UNK	UNK
γδ T	+	epiWAT (HFD)	UNK	UNK	UNK	UNK	UNK

+, pro-inflammatory; −, anti-inflammatory; #, multi-functional;

* , inconsistent results;

*/, not discussed in this review; UNK, unknown*.

## Immune Cell Crosstalk Regulates Obesity-Associated Inflammation

### Role of APCs in Obesity-Associated T Cell Inflammation

Current investigations on interactions among immune cells in obesity have mainly focused on standard APC-T cell interactions, as identified in classical “signal 1 and signal 2” splenocyte and PBMC studies, but potentially occurring within the AT as well due to lymphocyte recirculation. Overall, DCs, B cells, macrophages and adipocytes have reported APC activity in AT as discussed superficially above and in more detail below, but there is no consensus on whether any of these APC subsets dominates CD4^+^ T cell activation and thus T cell inflammation in obesity-expanded AT (7, 8). Analysis of HFD-fed mice indicates that B cell-specific signatures (B220 and CD19) are maximal after 3 weeks of HFD if mice are started on diet shortly after weaning (40, 74). The timing of increased numbers and frequencies of macrophages after the start of HFD is delayed relative to B cells, hinting that B cells may provide an initial burst of APC activity, while macrophages and DCs may at least partially take over later, all in the context of MHC Class II upregulation on adipocytes 2 weeks after start of HFD (107). In addition to uncertainties over specific cell types that provide the MHC Class II molecules needed for CD4^+^ T cell activation, numerous costimulatory molecules on the APC that strengthen, sustain or ameliorate TCR activation have also been implicated in obesity-associated T cell inflammation. Interactions between CD40/CD40L, CD80/86/CD28, ICOS/ICOSL, PD-1/L1&2, and OX40/OX40L among others were reported to regulate inflammation in obesity (108, 109). Each of these regulatory pairs directly impacts T cell proliferation and cytokine production under a variety of conditions, although the relative contribution in contexts more physiological than simplistic knockout/blockade/activator studies will require significantly more subtle analyses to go beyond the well-established finding that almost any method that decreases inflammation also decreases obesity and/or metabolic dysfunction in animal models.

### Role of B Cells in Obesity-Associated T Cell Inflammation

B cells present antigens to T cells and can activate both macrophages and DCs. Although B cells secrete relatively small concentrations of obesity-associated cytokines, their role in inflammation is amplified by a unique ability to concentrate antigen though epitope-targeted uptake/processing to regulate T cell function. The regulatory role of B cells in obesity-associated T cell inflammation was first shown in mice under HFD conditions. Obese B cell-knockout mice had fewer Tregs in blood, AT and spleen (110), which indicated that, as in other systems, B cells controlled anti-inflammatory Tregs in obesity/T2D (74). B cell-null mice on a HFD also had less frequent IFN-γ-producing CD4^+^ and CD8^+^ T cells and overall less IFN-γ production by VAT, suggesting additional mechanisms may impact the outcome of B-T crosstalk in obesity (73). Together these changes correlated with improvements in metabolic health in B cell-null compared to wild-type mice. In stark contrast to most obesity-associated inflammatory queries, work on the role B cells play in human T2D inflammation are somewhat more mechanistically advanced than mouse studies, which have mainly used knock-outs as a relatively blunt tool. Circulating human B cells directly regulate a bioinformatically derived Th17 “profile” that discriminates inflammation in samples from T2D and normoglycemic obese people. The Th17-targeted pro-inflammatory mechanism of B cell regulation is unique to cells from the T2D milieu, and contrasts with the more general support of Th17s by myeloid cells in samples from both types of subjects. B cells and T cells must touch, or at least be in close approximation, for T2D-potentiated Th17 activation, suggesting costimulatory molecule involvement that is specific to B cells over, in this case, monocytes. Complementary demonstrations that IL-17A and IL-22 production were significantly lower following stimulation of B cell-depleted PBMCs of T2D subjects independently supported the idea that B cells activate Th17s in T2D (42, 74). These studies did not rule out the possibility that short-term activators like reactive oxygen species, known to be increased in T2D, may mediate T2D-limited B/T cross-talk. Exploration of these outcomes in mice awaits generation of a mouse model that more closely recapitulates the Th17 profile characteristic of humans.

### Role of Myeloid Cells in Obesity-Associated T Cell Inflammation

Macrophages and DCs are at extreme ends of a continuous spectrum of myeloid cells, and they are amongst the most potent APCs in obesity despite long-term disagreement on defining myeloid subsets based on surface markers. Myeloid APCs, unlike B cells, have a limited ability to specifically concentrate antigens. DCs in the AT likely impact Th17 maturation by producing IL-1β, IL-6, IL-23, and TGF-β (40, 111, 112), and Th1 polarization by producing cytokines like IL-12 and IL-18, albeit from different DC subsets (111). Cytokine-mediated crosstalk between myeloid cells and T cells is also regulated upstream by free fatty acids that increase TLR2 and TLR4 expression on DCs and thereby enhance secretion of IL-23 and IL-12 in response to obesity-associated stress signals (113). DC-derived cytokines can also promote inflammatory comorbidities of obesity/T2D, including psoriasis-like skin inflammation in mice (113). DC cytokines complement the more widely appreciated role of DCs as potent APCs, and directly activate T cell inflammation in AT as discussed above. Roles for DCs in obesity-associated hepatic inflammation are also suggested by demonstrations that DCs have significantly higher expression of the costimulatory molecule CD86 in liver of obese mice compared with lean controls (114).

Although macrophages are important producers of obesity-associated cytokines, their APC function is somewhat specialized in the HFD model of mouse obesity/T2D. In this model, AT resident macrophages initially interact with naïve CD4^+^ T cells via MHC Class II molecules in the acute inflammatory milieu. Later, an established inflammatory environment recruits CD11c^+^ macrophages to AT that in turn induces conventional T cells to proliferate and thereby sustain adaptive immune-derived inflammation in AT (115). Dynamic interaction between CD11c^−^ or CD11c^+^ macrophages with CD4^+^ T cells was found to control AT inflammation through macrophage-expressed MHC Class II molecules (115). Regardless of all the details elucidated by these studies, the lack of defined obesity-associated antigens remains a barrier to make the most of these findings clinically relevant. Effects of obesity/T2D on the TCR repertoire in the AT add more potential layers of complexity (29).

### Adipocyte Coordination of Obesity-Associated Inflammation

Large adipocytes from HFD-fed mice highly express MHC Class II, which activates CD4^+^ T cells and promotes Th1 proliferation and IFN-γ production in AT (116). Adipocyte-specific MHC Class II knockout increased AT Treg numbers by decreasing IFN-γ production in obesity (107). Adipocyte APC activity therefore combines with adipokine secretion, which further activates and modifies the cytokine profile of partnering T cells. Adipocytes also express high amounts of the non-traditional MHC CD1d, which enables them to act as lipid presenting cells and activate iNKT cells (64, 117), as discussed above. *In vitro* studies demonstrated that physical interaction between adipocytes and iNKTs regulate IL-2 secretion from activated iNKTs to further modulate inflammation through effects on general T cell survival/activation. Given that a unique network of lipid metabolites is generated under metabolic stress in AT, CD1d-mediated lipid presentation may regulate a unique corresponding iNKT cytokine profile during obesity. However, not all studies agree that adipocytes activate CD4^+^ T cells through APC activity (115).

The nature of the immune cell-activating lipid antigens produced by stressed adipocytes remains unknown, but intracellular processing and endocytosis of lipid metabolites generated during obese stress may provide insights toward understanding how immune cell subtypes are regulated in AT. The ER-Golgi pathway and endosomal/lysosomal pathway assemble and traffic glycolipids that could be cognate antigens for AT-resident immune cell populations. Alternatively, endocytosis of metabolites generated in obesity may mediate tissue-specific TCR activation by either professional or non-professional APC activity. Recent work showed that the GTPase Rab4b, a molecular switch that governs endocytosis, decreased in AT-associated T cells of both obese humans and mice. T cell-specific Rab4b knockout increased the production of IL-17A, IL-6, and IL-1α in the epiWAT which resulted in an early increase in AT Th17 cells in combination with Treg loss. The authors concluded that Rab4b-dependent TCR trafficking to the plasma membrane prevents differentiation of pro-inflammatory Th17 cells in the AT (118, 119).

## Immune Cells in Obesity-Associated Cancers

**Figure 2:**
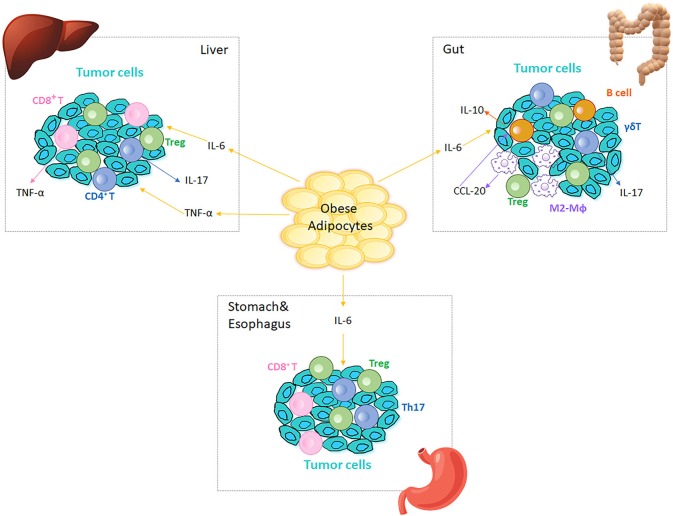
Immune cells in obesity-associated cancers. Obese AT adjacent to cancer sites generates IL-6 and TNF-α, which are inflammatory initiators of tumor cell migration in different tissues/organs. Tregs are present at tumor microenvironment in liver, gut, stomach and esophagus. In liver, IL-6 and TNF-α increase to promote cancer cell proliferation, which in turn recruits CD4^+^ and CD8^+^ T cells to the tumor microenvironment. CD4^+^ T cells produce IL-17 and both CD4^+^ and CD8^+^ T cells produce TNF-α, which further augment the inflammation in liver and aggravate HCC. In colitis, IL-6 regulates intestinal macrophages (Mϕ) polarization to M2-like phenotypes (M2-Mϕ) that produce CCL-20 and recruit CCR6-expressing B cells to the tumor microenvironment. B1 B cells can also polarize macrophages by generating IL-10 in response to the alterations in gut microbiota. CCR6-expressing γδ T cells produce IL-17, which promotes colonic inflammation and deteriorates colorectal cancer. Omentum-derived CD8^+^ T cells are enriched in the stomach and esophageal tumor microenvironment, and Th17 responses are triggered in the presence of *Helicobacter felis* in stomach.

The epidemiological evidence linking obesity to cancer is compelling (120). Cancers in GI tract, including colon, liver, esophagus, and stomach, are associated with obesity likely due to anatomical proximity: colorectal cancer directly associates with mesenteric adipose tissue, and hepatocellular carcinoma develops in an organ directly embedded with VAT (120). Oesophagogastric and gastric carcinoma are directly exposed to excess dietary fat intake and digestion products (121). Obesity-associated changes in immune cell function in the tumor microenvironment may mechanistically explain, at least in part, the connection between obesity and certain cancers (120, 121). The chronic inflammatory status in obesity disrupts tissue homeostasis, hampers protective responses, and results in tissue hyperplasia or necrosis in both human and murine systems to perhaps trigger the first steps of obesity-associated tumorigenesis (120, 122–124). Classical obesity-induced pro-inflammatory cytokines, which mainly originate from immune cells and include TNF-α, IL-6, and TGF-β, participate in tumor cell proliferation and invasion, and may subsequently contribute to tumor progression (125, 126). Phenotypic changes in AT macrophages, CD4^+^ and CD8^+^ T cells are closely related to tumor growth and metastasis at sites adjacent to AT (127). The hypoxic nature of tumors may also promote obesity-associated cancers, since cells in the center of solid tumors are even more anoxic than hypertrophied adipocytes in obesity (10). Tumor hypoxia induces HIF-1/NF-κB signaling, triggers expression of pro-inflammatory genes, and promotes the highly cytokine-secreting phenotype of cancer (128–131). In turn, the inflammatory cells augment tumor vascularization by producing vascular endothelial growth factor (132). A combination of obesity and tumor-associated hypoxia may multiply the impact of each individual pro-inflammatory process. Finally, some studies indicate a link between obesity and bone marrow-associated cancers such as bone tumors and skeletal metastasis (133, 134) that may further impact development of immune cells and subsequent responses. Figure 2 outlines roles for immune cells in obesity-associated cancers as detailed below.

### Obesity-Associated Inflammation and Colorectal Cancer

Obesity-induced inflammation may confer additional cancer risk beyond obesity *per se* (121). For example, persistent obesity-related inflammation can induce colorectal cancer mutagenesis through reactive oxidative damage and epigenetic silencing to promote cancer growth (121, 135). Obesity increases IL-6 production (136–139), which in turn regulates the tumor microenvironment of colitis-associated colorectal cancer by shifting intestinal macrophage polarization to the tissue regenerating M2-like macrophages that functionally overlap with tumor-associated macrophages in mouse models (140). M2-like macrophages produce CCL-20, which recruits CCR6-expressing B cells and γδ T cells, perhaps to the tumor environment. Colon B1 B cells can also polarize macrophages to an M2-like phenotype by producing IL-10 (in response to the alterations in the gut microbiota) to blunt tumor-fighting inflammation (140). Apart from a link to M2-like macrophage regulation, CCR6-expressing γδ T cells produce pathogenic IL-17, which aggravates colonic inflammation (and thereby perhaps tumor clearance), albeit in a mouse with a deleted TCRα gene (141). Blocking recruitment of B cells and γδ T cells to the colon alleviated colitis and consequently suppressed the development of colorectal cancer. Although γδ T cells can promote colonic inflammation, they also play a role in tumor immunosurveillance, consistent with the demonstration that fewer γδ T cells in colon submucosa due to obesity-associated hypercholesterolemia sabotaged the initiation of cytotoxic effects toward tumor site (142). Based on the multifaceted roles of IL-6, M2-like macrophages and γδ T cells in obesity and associated cancers, the importance of these as either biomarkers or mediators of clinical treatments remains tentative.

### Obesity-Associated Inflammation and Hepatocellular Carcinoma

Obesity-associated hepatic inflammation is characterized by various cytokines and adipokines that have been generally implicated in carcinogenesis (143). In a long-term choline-deficient high-fat diet (CD-HFD) murine model of HCC, cancer developed after 12 months, concomitant with robust increases in activated CD4^+^ and CD8^+^ T cells, but absent changes in liver-associated CD19^+^ B cells. Liver-associated CD8^+^ T cells secreted TNF-α, and CD4^+^ T cells produced IL-17 in this model. Intrahepatic NKT cells and Tregs also increased, recapitulating findings in NASH and HCC patients, but also demonstrating a potentially mixed infiltration of pro- and anti-inflammatory immune cells (144). The clinical parallels of these processes in human colorectal cancer are not known.

### Obesity-Associated Inflammation in Other GI Carcinomas

In addition to gut and liver cancers, oesophagogastric adenocarcinoma (OAC) and gastric carcinoma are also influenced by obesity-induced inflammation (60, 145, 146). Effector memory CD8^+^ and CD4^+^ T cells are enriched in the omentum, a fold of peritoneal tissue that connects the stomach to various other abdominal organs, and a significant VAT depot of OAC patients (145). Animal model studies inspired by this clinical observation showed that blockade of the chemokine receptor CCR1 inhibited T cell infiltration into the omentum and reduced omentum inflammation (146). The weakness in leveraging these observations into clinical advances is that front line cancer treatments like chemotherapy and radiation cause chemokine fluctuations that may reduce efficacy of chemokine-targeting anti-inflammatory strategies in OAC. Obesity also accelerated gastric carcinoma development in response to *Helicobacter felis* in mice, at least in part by promoting Th17 responses in the stomach, accompanied by macrophage infiltration into the VAT (147).

Although the work above tested the hypothesis that obesity induces a chronic low-grade inflammation to generally exacerbate cancer progress, recent clinical studies showed improved efficacy of checkpoint blockade immunotherapy, which should increase T cell co-stimulation and thus inflammation in obese compared to lean patients. This unexpected outcome implied that a negative impact of obesity-associated inflammation on cancer somehow became a net positive under these treatment conditions (148). Attempts to clear tumors by inactivating protective Tregs or myeloid cells to “release” anti-tumor effector T cells and myeloid cells is similarly at odds with the possibility that overexuberant Th17 or Th1 T cells, absent the balancing influence of Tregs, characterize obesity and T2D while somehow failing to attack tumor cells. These apparent paradoxes suggest that important outstanding questions on the relationships amongst immune cell inflammation, obesity and cancer must be answered before clinical trials can incorporate an understanding of obesity-associated inflammation and cancer into new treatment approaches (149, 150).

## Concluding Remarks

Immune responses from classically delineated innate and adaptive immune cells combine for a tightly orchestrated cacophony that maintains systemic homeostasis. Cytokines are messengers that connect a complicated network of tissue-specific immune cells to maintain organismal health. With the progression of obesity and obesity complications, the peaceful co-existence is disrupted: leukocyte subpopulations ramp up cytokine production unevenly, producing dominant players amongst others that may be compromised but may also more simply act as bystanders. The resolution signals from certain immune cells to counter the chronic low-level inflammation in tissues during obesity are gradually impaired with the reciprocal increase in systemic inflammation (52, 62, 151, 152). Since the majority of current conclusions are generated from murine models with limited testing in human cells, it is challenging and risky to immediately translate most concepts highlighted herein to moderate the many effects of obesity-associated inflammation amongst metabolic comorbidities in the clinic. Unfortunately, a physiologically useful cutoff value for “inflammation”, or a panel of markers for defining inflammatory stages of obesity, is not available. Due to the chronic, low-grade character of obesity-associated inflammation, circulating correlates may not be detectable in patients until a dangerously late or irreversible state develops, and may require analyses far beyond typical clinical work-ups. Therapeutic interventions may target costimulatory molecules and coinhibitory pathways which are selectively acting under pro-inflammatory conditions (109, 153–155); targeting iNKT or γδ T cells could also be a choice (156, 157), as may be targeting of specific fatty acid metabolites (46). All of these possibilities are theoretically fraught with pleiotropic actions of the cells and molecules targeted. More comprehensive and combinatorial analyses of inflammatory cascades and reciprocal regulation among immune cell subsets in obesity are desperately needed toward the long-term goal of developing innovative and safe therapeutics to treat the ever-lengthening list of obesity comorbidities.

## Author Contributions

All authors listed have made a substantial, direct and intellectual contribution to the work, and approved it for publication.

### Conflict of Interest Statement

The authors declare that the research was conducted in the absence of any commercial or financial relationships that could be construed as a potential conflict of interest.
